# Influence of Starch on the Structure–Properties Relationship in Polyethylene Glycol/Polycaprolactone Diol Polyurethanes

**DOI:** 10.3390/polym14153184

**Published:** 2022-08-04

**Authors:** Jhoan F. Cespedes, Said Arévalo-Alquichire, Luis E. Diaz, Manuel F. Valero

**Affiliations:** 1 Energy, Materials and Environmental Group, GEMA, Faculty of Engineering, Universidad de La Sabana, Chía 140013, Colombia; 2 Master Program in Design and Management Process, Faculty of Engineering, Universidad de La Sabana, Chía 140013, Colombia; 3 Department of Ophthalmology, Schepens Eye Research Institute of Mass Eye and Ear, Harvard Medical School, Boston, MA 02114, USA; 4 Bioprospecting Research Group, GIBP, Faculty of Engineering, Universidad de La Sabana, Chía 140013, Colombia

**Keywords:** zwitterionic starch, potato starch, polyurethane, hard segments, soft segments, PCL, PEG, PE

## Abstract

Improvements in the antithrombogenicity activity of biomaterials for cardiovascular applications are necessary to meet the demand for vascular grafts in the world. Zwitterionic compounds tend to be used due to their anti-fouling properties, which reduce platelet adhesions and protein absorptions. Therefore, in this research, potato starch (AL-N) and zwitterionic starch (AL-Z) (obtained by Williamson etherification) were included as fillers in polyurethane (PU) matrices from polycaprolactone diol (PCL), polyethylene glycol (PEG), pentaerythritol (PE) and isophorone diisocyanate (IPDI) in order to study their effect in terms of their physicochemical, mechanical and thermal properties. We conducted our evaluation using attenuated total reflectance Fourier transform infrared spectroscopy (ATR-FTIR), contact angle analysis, swelling behavior, thermogravimetric analysis (TGA), tensile/strain analysis, scanning electron microscopy equipped with energy dispersive X-ray spectroscopy (SEM-EDS), dynamic mechanic analysis (DMA), differential scanning calorimetry (DSC), and X-ray diffraction (XRD). The results showed that AL-N and AL-Z modified these properties, where AL-N improved tensile strength, and AL-Z increased the hydrophilicity of polyurethanes matrices; additionally, AL-N had interactions with the soft segments, and AL-Z had interactions with the hard segments. Finally, both fillers reduced the degree of crystallinity and did not affect the thermal stability of polyurethanes.

## 1. Introduction

Composite polymers tend to be used in cardiovascular applications [1] due to their versatility in terms of their mechanical, thermal, and physicochemical properties, which are achieved by mixing a disperse phase (filler) and a continuous phase (polymeric matrix) [1,2]. Several investigations with different polymeric matrices have been conducted to obtain new composite materials for cardiovascular implants, especially with alginate, poly(D,L-lactic-co-glycolic acid) (PLGA), polytetrafluoroethylene (PTEF), polyethylene terephthalate (Dacron), and polyurethane (PU) [3,4,5,6,7,8]. Between these polymeric matrices, polyurethanes are listed as the first candidate for the production of vascular grafts owing to their better compliance, biocompatibility, mechanical properties, and adaptation to different manufacturing technologies [9,10].

Polyurethane is a group of polymers synthesized by a polycondensation reaction between an isocyanate and polyol [11]. During polycondensation, urethane groups are formed and begin to interact with polar groups present in polyols. As a consequence, a hard crystalline domain appears that is held together due to hydrogen bonding. In contrast, interactions between the polyols are weak, and a soft domain is produced [9,12]. The mechanical properties of polyurethane can be modified by the change of soft and hard segments controlling the flexibility and hardness of the polymer. Thus, graft compliance can be optimized by varying concentrations of those segments [12]. Polyester and polyether blends have been studied to improve the biological and mechanical performance of polyurethanes. In fact, polycaprolactone-diol (PCL) has shown promising results referred to as biocompatibility and mechanical properties. It is associated with the interactions of hard segments. Moreover, polyethylene glycol (PEG) is often used to provide flexibility and antithrombogenicity activity [12,13]. Despite this, blends of these polyols have been used in the synthesis of polyurethane for biomedical applications [14,15]. Recently, investigations have shown that the inclusion of fillers in polymeric matrices constituted by different polyols can improve mechanical and thermal properties [16,17].

Certain studies have reported the improvement in the mechanical properties of polyurethane with the addition of natural fillers such as nanoparticles of chitosan, cellulose, and starch. Villani M et al. [18] studied the effect of loading TiO_2_, chitosan, and silver in polyurethane, finding that chitosan and silver could be used as fillers for medical applications. Another investigation conducted by Arevalo F.R et al. [19] concluded that adding chitosan as a filler in a polyurethane matrix synthesized from castor oil does not affect mechanical and thermal properties but also improves the L929 cell’s viability. Furthermore, Hormaiztegui M et al. [20] added cellulose nanocrystals to the polyurethane matrix, finding that the mechanical and thermal properties were reinforced. Around starch particles, Gaaz T et al. [21] loaded starch in a thermoplastic polyurethane matrix, reporting that fillers increase tensile strength by 17% with 1.5% starch.

Starch has been widely used in biomaterials and has potential applications, especially in cell seeding, tissue engineering, and implants for bone replacement [22]. This polysaccharide composed of amylose and amylopectin [22,23] is the second largest natural polymer found in plants as granules after cellulose, making it an extensive source of low-cost polymers. Polysaccharide is attractive for biomaterial due to their properties such as their biodegradability and because its degradations products are nontoxic; additional benefits include their biocompatibility, hydrophilicity, high chemical reactivity, and polyfunctionality [24,25]. However, starch has poor mechanical strength, causing it to need to be modified chemically or blended with other polymers [24].

One modification that can make starch a better material for cardiovascular applications is the inclusion of a zwitterionic moiety. Zwitterions are molecules that have mixed charge pairs, which confer super hydrophilicity and non-fouling properties [26]. Using a Williamson etherification reaction, Wang et al. [27] obtained zwitterionic starch from sulfobetaine and potato starch, which include the zwitterion moiety in the anhydroglucose unit and characterized the protein adsorption, cytotoxicity, and cell adhesion. They concluded that starch-modified material has protein resistance, good biocompatibility, and can resist cell adhesion.

From the trajectory of studies in the cardiovascular applications of polyurethane composites to the best of our knowledge, investigations have not been carried out on the relationship between starch (natural and modified with sulfobetaine) and soft and hard segments in polyurethane matrices, aside from its influence on mechanical and thermal properties. Therefore, this work describes the relationship between the concentrations of starch in polyurethane matrices synthesized from polycaprolactone diol (PCL), polyethylene glycol (PEG), and pentaerythritol (PE), with different soft and hard segments on physicochemical, mechanical, and thermal properties and the interactions of starch with those segments. We find that the interaction of starch with polyurethane matrices depends on its functional groups. Moreover, these interactions modify the mechanical and physicochemical properties to a significant degree.

## 2. Materials and Methods

### 2.1. Materials

Polycaprolactone diol (PCL-diol, Mw~2000), isophorone diisocyanate (IPDI), potato starch (soluble), N,N-dimetylformamide (anhydrous, 99.8%) (DMF), 1,3-propanesultone (PS) (98%), 3-dimethylamino-1-propyl chloride hydrochloride (96%) (CDMAP*HCl), and dichloromethane (anhydrous, ≥99.8%) were supplied by Sigma-Aldrich (St. Louis, MO, USA). Polyethylene glycol (PEG, Mw~1000) and sodium hydroxide were provided by Merck KGaA (Darmstadt, Germany). Pentaerythritol (PE) was obtained from Alfa Aesar (Heysham, UK) and glacial acetic acid (99.7%) was obtained from Central Drug House (P) Ltd. (Vardaan House Ansari Road, Daryaganj, New Delhi, India).

### 2.2. Synthesis of Zwitterionic Starch

The obtention of zwitterionic starch was carried out using Williamson etherification. Briefly, 4.02 M CDMAP*HCl in distilled water was neutralized with the addition of 40% NaOH (keeping the stoichiometry relationship) and the oil phase (CDMAP) was decanted. In addition, CDMAP was added to PS (1.36 M in dichloromethane), reacting for 24 h at 28 ± 2 °C in a nitrogen atmosphere. The product that was precipitated (3-dimethyl (chloropropyl) ammonium propanesulfonate or DCAPS) was washed three times with dichloromethane, dried, and stored under vacuum, and its obtention was confirmed by ^1^H NMR [27]. Moreover, potato starch was activated with 25% NaOH, keeping the mol relationship of the NaOH/anhydroglucose unit at 1:2.5. DCAPS (2.46 M in distilled water), which reacted with the activated potato starch (2.19 M in water distilled) at 55 ± 2 °C for 12 h. The product in the solution reaction was neutralized with acetic acid glacial at a pH of 7 and precipitated with methanol, and we washed the solution reaction three times (Figure S1) [27,28]. The size of zwitterionic starch was reduced by pulverizing it in a mortar and sieving it in a 90 µm mesh.

### 2.3. Chemical Structure, Thermal and Morphology Characterization of Zwitterionic Starch

The obtention of zwitterionic starch was evaluated by Fourier transform infrared spectroscopy using diamond attenuated total reflection (ATR-FTIR) and proton nuclear magnetic resonance (^1^H NMR). ATR-FITR was taken with a Cary 630 FTIR spectrometer (Agilent, Santa Clara, CA, USA), recording in a range of 650 cm^−1^ to 4000 cm^−1^ with an average of eight scans and a resolution of 2 cm^−1^. ^1^H NMR was measured with a Bruker Avance III spectrometer of 400 MHz (Billerica, MA, USA), using 32 scans and D_2_O as solvent at 25 °C. The degree of substitution was obtained from equation 1, using ^1^H NMR integrals data from the peaks “c” (∫c) and “C1” (∫C1) corresponding to the resonance of hydrogens of the quaternary ammonium group in the sulfobetaine moiety and carbon 1 in the anhydroglucose unit [27,28].

DS = ∫c/(∫C1 × 6)(1)

Thermal characterization was carried out by thermogravimetric analyses (TGA) and differential scanning calorimetry (DSC). TGA measured the thermal stability of zwitterionic starch in two steps under a nitrogen atmosphere (100 mL/min) using a TGA/DSC 1 (Mettler Toledo, Columbus, OH, USA). First, water from modified starch was removed at 106 °C for 1 h. Then, samples were heated at 10 °C/min until 600 °C. DSC was taken with DSC 3+ (Mettler Toledo, Columbus, OH, USA), using a nitrogen atmosphere (100 mL/min) in a range of −70 °C to 150 °C with a ramp of 5 °C/min [29].

The surface morphology of zwitterionic starch was studied by scanning electron microscopy (SEM), using a TESCAN LYRA3 (Brno, Czech Republic) [30].

### 2.4. Synthesis of Polyurethane Composites

Polyurethanes were synthesized by the prepolymer method [31]. First, melted polyols (PEG and PCL) at 110 °C reacted with IPDI at 70 °C and 300 rpm for 15 min, obtaining a prepolymer. Second, a solution of PE 0.497 M in DMF at 110 °C was made, and we added that solution to the prepolymer and carried out the experiment at 70 °C and 300 rpm for 15 min, keeping a mol relationship of 1:1 NCO/mol OH [12,31]. The addition of starch (native and zwitterionic) was made after crosslinking with PE and homogenizing the powder in the media at 70 °C and 300 rpm for 15 min [31]. Films of polyurethane composites were obtained when the composite was poured into a stainless steel mold and cured at 110 °C for 12 h [32]. The composition in the weight of polyols and starch (native and zwitterionic) is exhibited in Table 1, where composition P1 corresponds to 5% PEG—90% PCL—5% PE, composition P2 corresponds to 45% PEG—45% PCL—10% PE and composition P3 corresponds to 46.25% PEG—46.25% PCL—7.5% PE.

### 2.5. Physicochemical Characterization 

Polyurethane composites chemistry was described by ATR-FTIR with the Cary 630 FTIR spectrometer (Agilent, Santa Clara, CA, USA) in a range of 650 cm^−1^ to 4000 cm^−1^ [30]. Water absorption was studied by the weight of the material before (W0) and after soaking in distilled water (WSx) at different times. Four points on the first day and three points per day in the next three days were recorded [32,33]. Equation (2) was used to determine the percentage of water absorption. The contact angle was measured by the sessile drop method, using the equipment MobileDrop (gh11, Krüss, Germany) and distilled water at 20 °C as a test liquid. The average measurement of the contact angle corresponds to ten values of each polymeric material [33,34].

% Water absorption = ((WSx − W0)/W0) × 100% (2)

### 2.6. Thermo-Mechanical Properties

The tensile/strain test was performed according to ASTM D638-1 in an EZ-LX (SHIMADZU, Kioto, Japan) with a 5 kN load cell and a deformation rate of 10 mm/min. This test was conducted in triplicate and each sample had dimensions of 40 × 6 × 3 mm (length × width × thickness) [34]. The morphologies of the interphase between fillers and polymeric matrices were studied by SEM in a TESCAN LYRA3 (Brno, Czech Republic) [30,35].

The storage modulus and the loss factor were evaluated by dynamic mechanical analysis (DMA) in a DMA 850 (TA Instruments, New Castle, DE, USA) with a frequency of 1 Hz and deformation of 1 mm. Samples of 20 mm × 5 mm × 1.5 mm (length × width × thickness) were heated with a ramp of 5 °C/min between −70 °C and 120 °C [31].

X-Ray diffraction (XRD) was measured in a X′PERT PRO MPD (PANalytical, Malvern, Worcestershire, UK) with a Cu α radiation at 45 kV and 1.54 A. The measurement range was between 5–70° (2 theta). Equation (3) was used to calculate the degree of crystallinity (DC) of composite materials, using integrals of XRD curves, where (Ic) is the area under the crystalline peak and (Ia) is the amorphous area [36].

DC = (Ic/(Ic + Ia)) × 100%(3)

Thermal transitions and thermal stability were studied by DSC and TGA. DSC was measured in a DSC3+ (Mettler Toledo, Columbus, OH, USA) under a nitrogen atmosphere (100 mL/min) between the range of −70 °C to 150 °C with a ramp of 5 °C/min. TGA was evaluated in a TGA/DSC (Mettler Toledo, Columbus, OH, USA) in two steps under a nitrogen atmosphere (100 mL/min). First, samples were exposed to an isothermal step at 106 °C for 1 h. Then, samples were heated at 10 °C/min in the range of 106 °C to 600 °C [12]. 

### 2.7. Statistics Analysis

Experiments were carried out using three independent replicates. The results are presented as a mean value ± standard deviation (SD). Data were analyzed by ANOVA of two factors to study the effect of the starch type (factor 1) and its concentration (factor 2) inside the PU matrix. Additionally, PU matrices without fillers were evaluated via ANOVA one-way. The assumptions of normality, homoscedasticity, and independence were validated. Significant differences were found by a Tukey test. A *p*-value of less than or equal to 0.05 was considered significant. 

## 3. Results and Discussion

**Figure 1:**
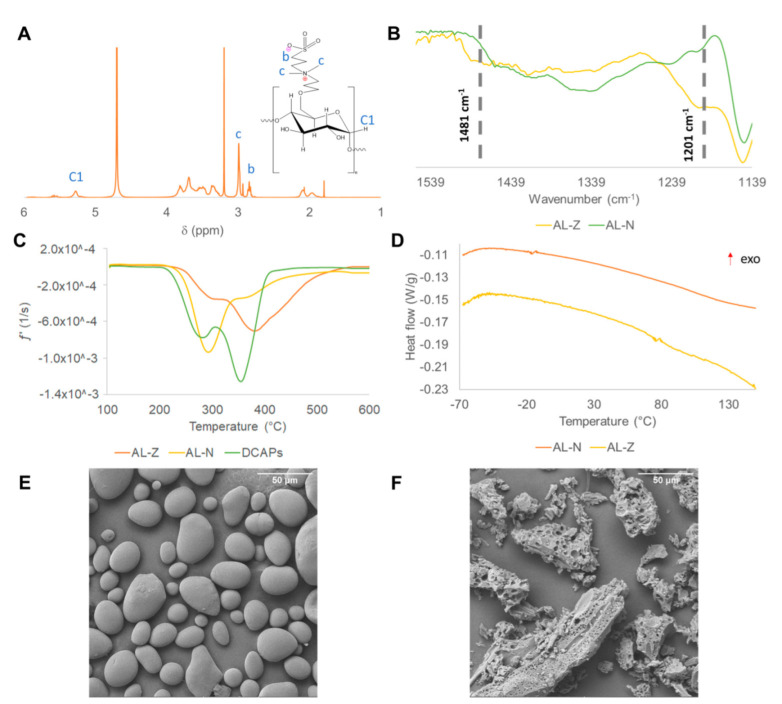
Characterization of starch (zwitterionic and native) by applying (**A**) ^1^H RMH, (**B**) ATR-FTIR, (**C**) DTG, (**D**) DSC, and SEM to (**E**) potato starch and (**F**) zwitterionic starch.

The obtention of the zwitterionic molecule (DCAPs) was confirmed by ^1^H RMN. Figure S2 shows the ^1^H RMN spectrum, in which five peaks were identified. Peak c (δ = 3.01) has major intensity due to it having a higher number of hydrogens (six H) with the same resonance than other groups in the molecule. These hydrogens are in the same electronic environment, near nitrogen with a positive charge (quaternary ammonium). The resonance of hydrogens near the sulfonate group corresponds to peak d (δ = 2.86). Additionally, peaks c and d appeared in the ^1^H RMN spectrum of zwitterionic starch (AL-Z) shown in Figure 1A, proving the modification of potato starch (AL-N) with the zwitterion (DCAPs). Another technique used to support the modification of starch was ATR-FITR. Comparing the FTIR spectra of AL-N and AL-Z shown in Figure S3, two new bands were identified in the AL-Z FTIR spectrum. These two new bands at 1481 cm^−1^ and 1202 cm^−1^ exhibited in Figure 1B are attributed to N-C and S=O stretching, confirming the inclusion of the zwitterionic moiety in the anhydroglucose unit of starch. These results are consistent with Wang et al. [27], who first synthesized DCAPS and then zwitterionic starch. DCAPS was used to modify potato starch by Williamson etherification. ^1^H RMN and FTIR spectra confirmed both products.

The degree of substitution (DS) defined as the average number of hydroxyl groups substituted per anhydroglucose unit [37], was estimated with equation 1 to find the inclusion of the zwitterionic moiety in potato starch. This value could vary in the range of 3 to 0, where 3 is the maximum and 0 is the minimum of zwitterion in starch. The areas of peaks c and C1 (hydrogen attached to carbon 1 in the anhydroglucose unit) of four replicas of synthesis (Figure S4) were used to calculate the DS, the value of which was 0.547 ± 0.03. This result is similar to the maximum DS of 0.46 obtained by Wang et al. [28], who studied the degree of substitution of the zwitterion moiety on potato starch at different temperatures, solvents, and mol relationships of NaOH and DCAPS with anhydroglucose unit.

The thermal stability of starches (AL-N and AL-Z) was measured by TGA. Thermograms (Figure S5) show the weight loss percentage of samples with the increment in temperature. The degradation steps of AL-N, DCAPS, and AL-Z were exposed in the derivative thermogram (DTG) exhibited in Figure 1C. All samples have two steps of degradation, where AL-N presents the first step between 218 °C–341 °C, which is related to the main degradation (pyrolysis) of amylose and amylopectin, and the second step in the range of 341 °C to 600 °C is attributed to the formation of carbon black [29,38]. The DTG of DCAPS shows the first step of degradation between 210 °C–308 °C, which is assigned to a Hoffman elimination of the quaternary ammonium [39] and the degradation of the sulfonate group. Additionally, the second step around 410 °C could be due to the breakup of the carbon chain [40]. AL-Z presented two steps of degradation, similar to AL-N and DCAPS, where the first step in the range of 220 °C–330 °C is associated with the degradation of the main chains (amylose and amylopectin) of starch-modified and the second step around 600 °C are related to the formation of carbon black. In the degradation steps of AL-Z, especially in the first step, there is an overlap between the degradation of the quaternary ammonium and sulfonate group with the break-up of amylose and amylopectin chains. Additionally, at the end of the first step of the degradation of AL-N, AL-Z lost less mass (15%) in comparison with AL-N, which lost 35% of its. This phenomenon is associated with an improvement in thermal stability due to the inclusion of sulfonate groups [41,42].

The thermal transitions of starches were studied by DSC. AL-N and AL-Z did not present any thermal transition (Figure 1D). Some investigations [43,44,45,46] have reported thermograms of potato starch, which presented a peak between 80 °C–110 °C related to gelatinization, a phenomenon that refers to a loss of structural order in amylopectin and amylose chains, depending on the content of moisture. Therefore, the peak absence in AL-Z and AL-N in Figure 1D after 80 °C indicated that samples do not present moisture and are thermally stable. 

Images obtained by SEM showed that potato starch (Figure 1E) has a spherical shape and smooth surface with a particle size of 24.4 ± 7.5 µm (*n* = 10). In contrast, AL-Z (Figure 1F) exhibited a porous surface and a non-uniform shape with a particle size of 88.5 ± 14.3 µm (*n* = 10), which is greater than AL-N.

**Figure 2:**
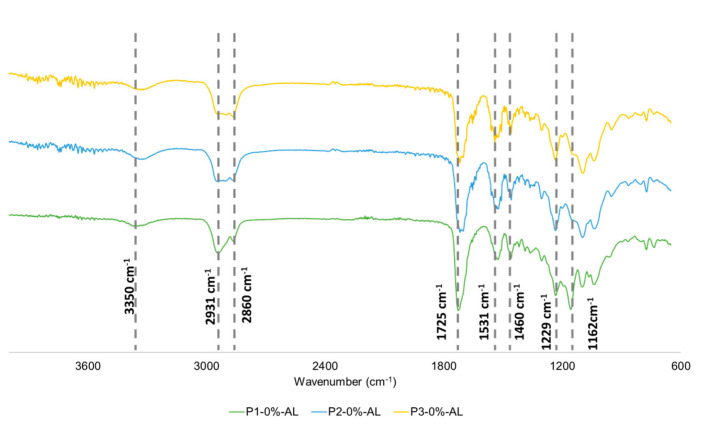
FTIR spectra of polyurethane matrices.

After the obtention of AL-Z, polyurethane composites were synthesized and evaluated by ATR-FTIR. Figure 2 shows FTIR spectra of polyurethanes P1, P2, and P3 without fillers, where FTIR bands in 3350 cm^−1^ (N-H stretching), 1725 cm^−1^ (C=O stretching), and 1531 cm^−1^ (N-H flexion) confirmed the obtention of polyurethanes. The presence of PCL and PEG segments in polyurethanes was observed in FTIR spectrums, in which the ester group of PCL is related to peaks at 1229 cm^−1^ (C-C(O)-O asymmetric stretching), and the increase in the intensity of the band at 1725 cm^−1^ (only in P1); moreover, the ether group is assigned to the band at 1162 cm^−1^ (C-C-O asymmetric stretching) [19,30]. The absence of the 2225 cm^−1^ peak assigned to the isocyanate group suggests that the IPDI completely reacted; that must be ensured due to the cytotoxic effect of that compound [31]. Finally, S. Arevalo-Alquichire et al. [12] acquired similar results for polyol compositions similar to the ones used in this study. 

**Figure 3:**
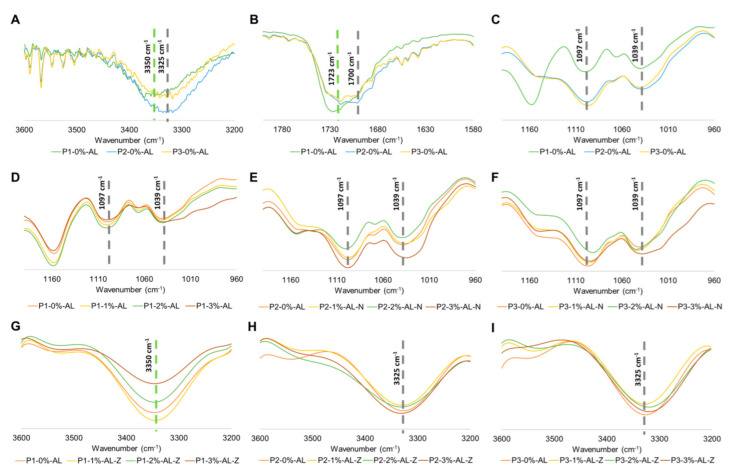
FTIR characterization of PU-starch composites. PUs matrices without filler in (**A**) the N-H region (**B**) C=O region (**C**) C-O-C region. (**D**–**F**) PU matrices with AL-N filler in C-O-C region. (**G**–**I**) PU matrices with AL-Z filler in N-H region.

The FTIR spectrum of polyurethane composites is shown in Figure S6. Intermolecular interactions between fillers and hard and soft segments of polyurethane matrices at the interface were evaluated by peaks at 3350 cm^−1^, 3325 cm^−1^, 1723 cm^−1^, 1700 cm^−1^, 1097 cm^−1^, and 1039 cm^−1^ (Figure 3). From Figure 3A, it could be seen that polymeric matrices P2 and P3 showed a displacement to the right side (3325 cm^−1^) in comparison with the peak for P1 (3350 cm^−1^). This phenomenon could be explained by the growth of hydrogen bonding between N-H (urethane group) and C-O-O (ether group) [47,48,49]. Furthermore, the intensity for this band in P2 increased in comparison to P1 and P3, which indicated an enlarged concentration of urethane groups [31]. These facts permit us to infer that the concentration of crosslinker in the polymeric matrix controls the number of urethane groups and the interactions in hard domains [31,47,49]. This statement is complemented by the behavior in Figure 3B,C, where a peak appears at 1700 cm^−1^ and increases the intensity at 1039 cm^−1^; this is related to the stretching of C=O (ester group) and C-O-C (ether group) with hydrogen bonding [48,50]. These results are consistent with Wongsamut et al. [47], who made polycarbonate-based thermoplastic polyurethane elastomers and studied the content of hard segments. They found that high concentrations of hard domains increase the number of hydrogen bondings.

Figure 3D–F exhibits a disruption at 1039 cm^−1^ for P1-3%-AL-N in comparison with P1-0%-AL. That band is related to the stretching of C-O-C of the ether group; thus, the enlargement of this band could be explained by the disturbance of hydrogen bonding between urethane groups and ether groups of polyethylene glycol [51], caused by the inclusion of potato starch at this concentration. Similar results were obtained by Malay O et al. [51], who made composites from silica nanoparticles and thermoplastic polyurethane urea (TPU), concluding that silica nanoparticles interact more strongly with soft segments than with hard domains.

Furthermore, the decrease in the 3350 cm^−1^ peak intensity with the increment in AL-Z concentration in the P1 matrix polyurethane (Figure 3G) could be attributed to a reduction in hydrogen bonds between N-H and C=O groups [49,52]. These results are consistent with the investigation conducted by Lee, H.S et al. [52], who studied the phase separation rate between soft and hard segments of polyurethanes at different temperatures, finding that an increase in peak intensity of 3330 cm^−1^ is related to an enlarged number of hydrogen bonds between N-H and C=O groups, and it indicated that hard and soft segment phase separations increased. From Figure 3H,I, AL-Z filler did not affect the hydrogen bonds between groups of hard domains in P2 and P3 polyurethane matrices.

The surface hydrophilicity of polyurethane composites was evaluated using the contact angle (Table 2). Polymeric matrices without filler did not have any significant change in their contact angle. However, other studies that included PEG in the polyurethane backbone [53,54,55] observed that a major concentration of this polyol reduces the contact angle value and then shows enlarged hydrophilicity. This tendency could be explained by the increase in the soft segment backbone polyurethane chains’ mobility, where polar groups (especially PEG) can migrate to the surface and reduce the interfacial energy [55]. Therefore, it could be inferred that the concentration of PEG in our polyurethane formulations was not significant enough to enlarge the mobility of chains in soft segments.

Moreover, the addition of the AL-Z filler caused a significant change in the contact angle of polyurethanes P2 and P3, increasing their hydrophilicity. That phenomenon could be explained by the inhibition of soft segment crystallization with the inclusion of AL-Z, which enables PEG to migrate to the surface. These results are analogous to the phenomena exposed by Kumar et al. [56], who attributed the increase in the contact angle to a strong intermolecular hydrogen bonding between the cellulose nanofiber (filler) and the polyurethane matrix, enlarging the crystallinity of soft segments and the migration of hydrophobic groups to the surface of composites. The improvement in the hydrophilicity of the composite material makes it more attractive for cardiovascular applications due to its anti-fouling property. This property is associated with a major hydrophilic surface with the capacity to form a tiny layer of water that prevents the adhesion of platelets and proteins, which induce thrombus formation [57]; therefore, composite materials with AL-Z are candidates for application in the cardiovascular field. AL-N filler in all PU matrices and AL-Z for P1 did not affect the contact angle to a significant degree. 

**Figure 4:**
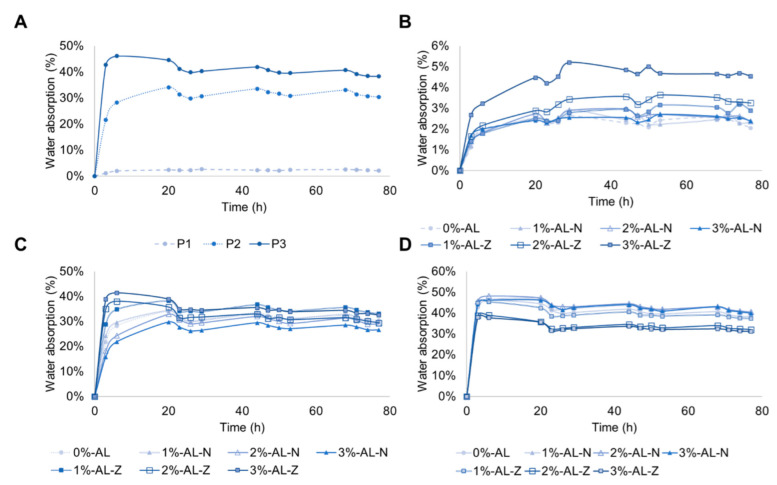
Water absorptions kinetics of (**A**) PU matrices without fillers, (**B**) P1, (**C**) P2, and (**D**) P3 composites.

The evaluation of the hydrophilicity of PU composites was complemented with the study of swelling behavior that was examined by water absorption. The weight change of samples related to the time they were soaked in water and the area under the curve are presented in Figure 4 and Table 2. From Table 2, it was observed that polyurethane matrices without filler exhibited statistical differences, in which P2 and P3 showed a larger area under the curve than P1. This area under the curve is associated with the capacity of polyurethane matrices to uptake water, considering the 80 h time frame. Then, P2 and P3 have a greater capacity to uptake water in comparison with P1. This result is assigned with the concentration of PEG, which enlarges the number of hydrophilic groups (ether groups) in the polyurethane backbone, improving the hydrophilicity of composites [54,58]. 

From Figure 4A–D, composite materials reach the maximum water absorption within the first 30 h. P1 polyurethane composites were the last materials to achieve the maximum water uptake (29 h) after P2 (20 h) and P3 (6 h). This behavior is associated with the crosslinking density and crystalline structure of segments, which modulate the resistance of the diffusivity of solvents inside the material [34,59]. Therefore, P1 composites take more time to reach a swelling equilibrium due to the semicrystalline PCL structure, followed by the P2 crosslinked structures and P3 materials, which contain the lowest PCL and PE (crosslinking agent) concentration in comparison with P2 and P1. Similar results were obtained by Fonseca et al. [59], who synthesized crosslinked polyurethanes from PCL and PEG, finding that an increase in crosslinked density and PCL concentration reduces the water uptake. On the other hand, adding fillers in polyurethane matrices did not have a different statistical significance.

The thermal stability of composites was evaluated by thermogravimetric analysis (TGA). Figure S7 shows the weight loss of materials with the increment in temperature. Figure S7A–D exhibit one step of degradation for all composite materials, and it occurs in the same interval of temperature (270–457 °C). This phenomenon could be explained by the degradation of starches that takes place between 260–400 °C [60], followed by the decomposition of polyurethane that happens between 310–380 °C [61]. The degradation of polyurethane occurs principally in three steps. First, urethane bonds and hard segments are broken up, followed by the crosslinker’s decomposition and, finally, the degradation of polyols due to the effect of temperature [31]. The temperature at which the maximum rate of degradation (Tmax) occurs (as reported in Table 2) did not show a statistically significant difference between polyurethane matrices with and without the inclusion of fillers (AL-N and AL-Z). Thus, the addition of starches in polyurethane matrices does not affect their thermal stability [60,61]. These results are consistent with the investigation carried out by Swamy et al. [60], who used starch as a filler, with starch loading concentrations of 5–25% in polymeric matrices, and concluded that the thermal stability of composites was not affected.

Results from the tensile/strain assay were reported in Table 2. Mechanical properties changed when the compositions of polymeric matrices were modified, in fact, P2 has greater tensile strength (4.76 ± 0.16 MPa) and secant module (3.21 ± 0.96 MPa) than P1 (σ = 2.4 ± 0.28, E = 1.99 ± 0.53) and P3 (σ = 3.13 ± 0.19, E = 1.52 ± 0.19). This could be explained by the change in hard segments, in which an enlarged concentration of hard domains causes an increase in tensile strength; in contrast, the enlargement of soft segments decreases the stiffness of polymeric material [31]. Similar results were obtained by Alquichire et al. [31], who made a design mixture of PEG, PCL, and PE and described a similar effect with the change in polyol composition and crosslinker.

**Figure 5:**
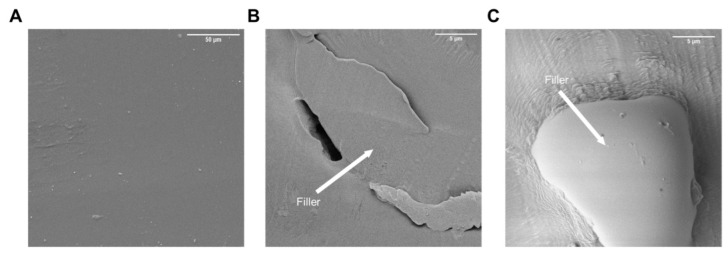
Representatives SEM images of P1 polyurethane composites at (**A**) 0%-AL, (**B**) 3%-AL-N, and (**C**) 3%-AL-Z.

The addition of starches (AL-N and AL-Z) influenced the mechanical properties. Tensile strength decreased to a significant degree when AL-Z was added to polymer matrices; additionally, the inclusion of AL-N did not result in a different statistical significance in tensile strength (except for P1-1% AL-N which increased), as can be noted in Table 2. This behavior is attributed to the thermodynamic compatibility between fillers and polyurethane matrices, which allude to the capacity to form hydrogen bonding at the interface, that intermolecular interactions adhere the surface of filler to the polymeric matrix, permitting the energy transmission on the boundaries of each material, then AL-Z acts as an inert material caused to not formations of hydrogen bonding at the interface with polyurethane in comparison with AL-N; therefore, starch modified with a zwitterion moiety creates defected areas that do not allow the transmission of energy when the composite is stretched, decreasing the tensile strength [51,62,63]. Additionally, the reduction in tensile strength of P1 composites filled with a concentration of AL-N that is greater than 1% wt indicates that the increase in the filler inside the polyurethane matrix permits formations of agglomerates and voids inside these agglomerates (Figure 5B), which reduce specific contact area at the interface filler–matrix [64]. These results are consistent with similar behavior reported in the literature for composite materials [51,62,63,64].

Furthermore, SEM images (Figure 5) and the elements weight composition of polyurethane composites (Table S1) obtained from EDS confirms the incorporation of fillers. Figure 5 points to the filler zone. From Table S1, the filler zone in P1-3%-AL-N had a major oxygen composition in comparison with the polyurethane matrix, which is related to potato starch. Additionally, polyurethane composite P1-3%-AL-Z in the filler zone showed sulfur, an element associated with the zwitterion moiety.

The reported tensile strength and strain at failure (%) of the femoral artery are 1 MPa and 76%, respectively. The composites synthetized in this work could be applied for the fabrication of synthetic vascular grafts since all the materials have a tensile strength and strain at failure above 1Mpa and 76%, respectively [65].

**Figure 6:**
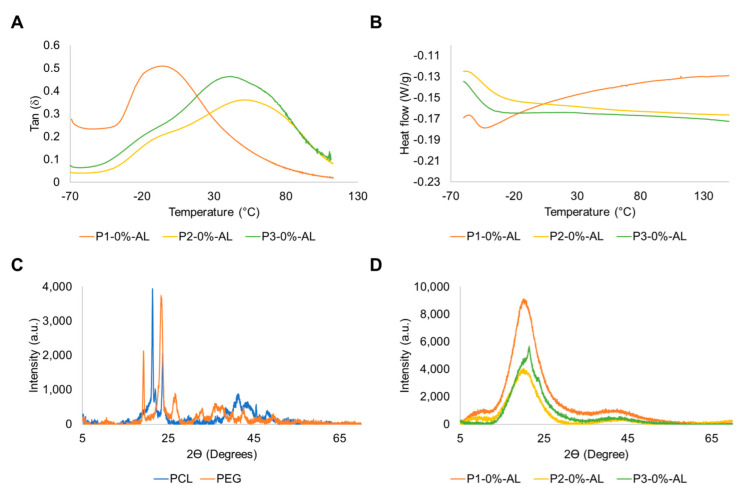
(**A**) Loss factor, (**B**) DSC, and XRD spectra of (**C**) polyols and (**D**) polymeric matrices without fillers.

The dynamic mechanical analysis recorded the loss factor (tan δ), storage modulus (E’), and loss modulus (E”). With the loss factor of matrices (Figure 6) and polyurethanes composites (Figure S8), the segregation of the hard and soft domains of materials was established. From Figure 6, it was noted that P1 had a homogeneous phase distribution due to the presence of one peak in tan δ. This result is due to the polarity of polycaprolactone diol (PCL), which permits ester groups to interact with urethane groups, making the generation of hydrogen bonding possible. This is consistent with the results reported by Asensio et al. [66], who studied the effect of different polyols (carbonates, esters, and ethers) on the separation of microphases (hard and soft segments) and their relationship with mechanical properties.

On the other hand, polyurethane matrices P2 and P3 showed a previous formation of a plateau form in the curve before the maximum value of tan δ. This result is caused by a partial mixture of soft and hard domains, principally due to the increase in crosslinking, which decreases the mobility of chains in the polyurethane backbone, and this phenomenon is attributed to an enlarged concentration of PE in the formulation of polymeric matrices. Furthermore, comparing polyurethane matrices P1 and P3, the increment in the concentration of PEG generated the segregation of soft and hard segments, produced by a lower polarity of PEG than PCL; additionally, this behavior is complemented by the reduction in the hydrogen bonding number that could be formed between a polar polyol such as PCL with urethane groups [31,66,67]. From Figure S8, it can be seen that adding filler in polymeric matrices did not affect the distribution of soft and hard segments compared with matrices lacking fillers.

The maximum storage modulus in the glassy region (Max E’) reported in Table 3 shows that the inclusion of AL-Z in polyurethane matrices P2 and P3 has the largest statistically significant value compared with polyurethane matrices with AL-N and without fillers. This could be explained by the theory of dual nanolayers formed around fillers, where a primary layer conformed by crosslinker chains is formed near particles, followed by a second layer that is assigned to chains that are less bounded [68]. Then, an enlarged storage modulus could be associated with an increase in interactions between chains that are crosslinkers in the first layer. Additionally, the glass transition temperature (Tg) is reported as the temperature which occurs at the maximum change of the storage modulus and is shown in Table 3. From these data, statically less Tg was noted for polyurethane composites P2 and P3 with AL-Z compared with P2 and P3 with AL-N and without fillers. This phenomenon is attributed to a reduction in the crystallinity region, which is related to improving chain mobility in bulk polymers. These results are consistent with those reported in the literature [21,69].

Differential scanning calorimetry (DSC) was used to study the thermal transitions of composites. Thermograms of polyurethanes are shown in Figure 6B and Figure S9 where materials exhibit only a glass transition. These glass transition temperatures (Table 3) were between −50.2 °C to −40.3 °C. Therefore, Tg from DMA and DSC indicates that composite materials are in the rubbery region at ambient temperature (12–20 °C) [70]. 

The XRD spectra of polyols and polyurethane matrices without and with fillers are shown in Figure 6C,D and Figure S10. From Figure 6C, it can be seen that PEG (peaks at 2ϴ = 19.3°, 23.5°, and 26.8°) and PCL (peaks at 2ϴ = 21.3° and 23.7°) are semicrystalline polyols; additionally, amorphous polyurethane matrices exhibited a peak between 19° < 2ϴ < 22° that can be assigned to crystalline regions conferred by PCL and PEG segments (Figure 6D). Variations in the concentrations of polyols change the intensity in the peak for polyurethane matrices, where P1 has the largest intensity due to a major concentration of PCL compared with P2 and P3. Furthermore, the inclusion of PE in polyurethane matrices caused a reduction in the intensity of this peak, and this could be attributed to a suppression of PCL and PEG crystalline segments. These results are similar to XRD spectra reported in the literature for polyurethanes obtained by PCL and PEG [31,71].

The crystallinity degree (DC) of polyurethane composites shown in Table 3 confirmed that the inclusion of AL-N and AL-Z in polyurethane matrices reduced the crystalline regions due to the disruption of interactions inside soft segments. Marand et al. [72] obtained analogous results with the inclusion of hydroxyapatite nanoparticles in polyurethane matrices with PCL-diol. 

## 4. Conclusions

Composite PUs were synthesized and confirmed by FTIR. Additionally, these materials were characterized in terms of their physicochemical, mechanical, and thermal properties, where the interaction of polyurethane matrices with fillers depends on the concentrations of soft and hard segments. FTIR showed that AL-N interacts with soft domains and AL-Z interacts with hard segments. As determined by contact angle and swelling assays, AL-Z increases the hydrophilicity of the polyurethane matrices P2 and P3. Tensile/strain assays show that AL-N improves tensile strength until 1% wt; on the contrary, AL-Z reduces that value for all PUs matrices. SEM images confirm the agglomerations of fillers and voids between these agglomerates. TGA showed that fillers did not affect thermal stability. Finally, DSC, DMA, and XRD showed that fillers reduce the crystallinity of PUs matrices, which is reflected in the reduction of Tg and DC. 

## Figures and Tables

**Table 1:** Composition of polyurethane composites.

PEG	PCL	PE	Starch	Sample *
5%	90%	5%	0%	P1-0%-AL
			1%	P1-1%-AL-(N ó Z)
			2%	P1-2%-AL-(N ó Z)
			3%	P1-3%-AL-(N ó Z)
45%	45%	10%	0%	P2-0%-AL
			1%	P2-1%-AL-(N ó Z)
			2%	P2-2%-AL-(N ó Z)
			3%	P2-3%-AL-(N ó Z)
46.25%	46.25%	7.5%	0%	P3-0%-AL
			1%	P3-1%-AL-(N ó Z)
			2%	P3-2%-AL-(N ó Z)
			3%	P3-3%-AL-(N ó Z)

* “AL” refers to starch (native and zwitterionic). “N” alludes to native starch and “Z” refers to zwitterionic starch.

**Table 2:** Physical and mechanical parameters of polyurethane composites, and ANOVA summary of water contact angle, area under the curve of water absorption, maximum temperature of degradation, and tensile strength and modulus.

PU	Starch	Concentration	Contact Angle (°) **	Area Under Curve (%Wt·h) **	Tmax (°C) **	Tensile Strenght (σ) (MPa) **	Module (E) (MPa) **
P1	AL-0%		103.7 ± 6.6 ^a^	1.60 ± 0.93 ^a^	420.29 ± 12.61 ^a^	2.4 ± 0.28 ^a^	1.99 ± 0.53 ^abcd^
	AL-N	1%	104.6 ± 4.7 ^a^	0.96 ± 0.72 ^a^	407.72 ± 11.16 ^a^	3.67 ± 0.18 ^b^	2.13 ± 0.08 ^abcd^
		2%	100.5 ±5.2 ^a^	2.52 ± 1.21 ^a^	415.78 ± 13.52 ^a^	2.34 ± 0.77 ^a^	2.25 ± 0.65 ^acd^
		3%	104.4 ± 1.8 ^a^	2.34 ± 0.52 ^a^	406.67 ± 5.52 ^a^	2.24 ± 0.27 ^a^	2.55 ± 0.23 ^a^
	AL-Z	1%	101.0 ± 1.3 ^a^	2.12 ± 1.43 ^a^	424.89 ± 8.10 ^a^	1.28 ± 0.14 ^c^	1.20 ± 0.024 ^bcd^
		2%	102.9 ± 1.5 ^a^	3.58 ± 0.44 ^a^	428.61 ± 2.04 ^a^	1.19 ± 0.039 ^c^	1.27 ± 0.12 ^cd^
		3%	110.3 ± 3.4 ^a^	3.68 ± 1.10 ^a^	429.22 ± 2.53 ^a^	1.15 ± 0.17 ^c^	1.46 ± 0.014 ^d^
	*p*-values						
	Starch		0.524	0.071	0.004 *	<0.001 *	<0.001 *
	Concentration		0.181	0.030 *	0.704	0.002 *	0.300
	Starch: Concentration		0.332	0.620	0.248	<0.001 *	0.070
P2	AL-0%		99.5 ± 6.5 ^a^	23.86 ± 6.76 ^a^	395.89 ± 5.59 ^a^	4.76 ± 0.16 ^a^	3.21 ± 0.96 ^a^
	AL-N	1%	99.5 ± 7.7 ^a^	24.17 ± 5.24 ^a^	398.83 ± 3.75 ^a^	4.08 ± 0.62 ^abc^	3.27 ± 0.72 ^a^
		2%	102.8 ± 3.6 ^a^	22.12 ± 2.79 ^a^	400.89 ± 2.59 ^a^	4.48 ± 0.90 ^a^	3.65 ± 0.81 ^a^
		3%	104.3 ± 1.4 ^a^	20.34 ± 2.28 ^a^	405.22 ± 3.14 ^a^	4.12 ± 0.13 ^abc^	4.05 ± 0.47 ^a^
	AL-Z	1%	83.4 ± 8.4 ^abc^	30.52 ± 2.19 ^a^	400.78 ± 8.68 ^a^	3.82 ± 0.47 ^abc^	3.20 ± 0.28 ^a^
		2%	57.9 ± 15.8 ^bc^	29.38 ± 4.19 ^a^	403.78 ± 2.45 ^a^	2.87 ± 0.34 ^bc^	3.05 ± 0.60 ^a^
		3%	68.5 ± 17.9 ^c^	30.90 ± 1.88 ^a^	402.83 ± 13.10 ^a^	2.59 ± 0.93 ^c^	3.04 ± 1.2 ^a^
	*p*-values						
	Starch		<0.001 *	0.004 *	0.823	0.002 *	0.220
	Concentration		0.028 *	0.615	0.200	0.003 *	0.888
	Starch: Concentration		0.006 *	0.257	0.902	0.042 *	0.677
P3	AL-0%		80.5 ± 17.6 ^ab^	32.28 ± 1.98 ^a^	401.11 ± 4.94 ^a^	3.13 ± 0.19 ^a^	1.52 ± 0.19 ^a^
	AL-N	1%	81.3 ± 11.8 ^ab^	36.57 ± 3.18 ^a^	406.44 ± 2.38 ^a^	2.87 ± 0.96 ^a^	1.37 ± 0.24 ^a^
		2%	77.1 ± 3.7 ^ab^	35.67 ± 2.45 ^a^	409.61 ± 3.67 ^a^	2.48 ± 0.35 ^acd^	1.32 ± 0.06 ^a^
		3%	84.5 ± 5.5 ^a^	31.85 ± 0.94 ^a^	412.11 ± 2.43 ^a^	1.78 ± 0.29 ^bcde^	1.12 ± 0.19 ^a^
	AL-Z	1%	57.5 ± 16.1 ^ab^	34.39 ± 2.48 ^a^	412.56 ± 2.59 ^a^	1.54 ± 0.29 ^cde^	1.51 ± 0.31 ^a^
		2%	43.1 ± 24.2 ^ab^	29.67 ± 5.64 ^a^	412.78 ± 0.09 ^a^	1.63 ± 0.37 ^de^	1.39 ± 0.19 ^a^
		3%	39.4 ± 14.8 ^b^	29.30 ± 10.86 ^a^	411.99 ± 9.78 ^a^	1.20 ± 0.30 ^e^	1.17 ± 0.21 ^a^
	*p*-values						
	Starch		<0.001 *	0.187	0.249	<0.001 *	0.448
	Concentration		0.128	0.378	0.003 *	<0.001 *	0.034 *
	Starch: Concentration		0.108	0.749	0.625	0.102	0.946

** Samples with the same letter do not have a statistically significant difference. Samples with a different letter have significant differences. * Effects are statistically significant (*p*-value < 0.05).

**Table 3:** Maximum storage modulus (Max E’), the glass transition temperature (Tg) from DMA and DSC, the degree of crystallinity (DC) of polyurethanes composite, and ANOVA parameters.

PU	Starch	Concentration	Max E’ (MPa) **	Tg from DMA (°C) **	Tg from DSC (°C)	DC (%)
P1	AL-0%		3004 ± 1508 ^a^	−32.89 ± 0.74 ^a^	−46.7	73.6%
	AL-N	1%	3042 ± 697 ^a^	−32.65 ± 0.39 ^a^	−47.7	63.7%
		2%	2703 ± 1357 ^a^	−33.02 ± 0.42 ^a^	−46.8	63.2%
		3%	3041 ± 729 ^a^	−33.61 ± 0.80 ^a^	−47.7	68.9%
	AL-Z	1%	3297 ± 127 ^a^	−33.75 ± 1.75 ^a^	−50.2	68.9%
		2%	3328 ± 388 ^a^	−34.83 ± 0.82 ^a^	−49.7	54.1%
		3%	3512 ± 647 ^a^	−35.80 ± 2.05 ^a^	−49.2	72.3%
	*p*-values					
	Starch		0.334	0.045 *	-	-
	Concentration		0.890	0.052	-	-
	Starch: Concentration		0.922	0.682	-	-
P2	AL−0%		4054 ± 274 ^a^	−24.94 ± 1.77 ^a^	−42.7	64.2%
	AL-N	1%	4708 ± 702 ^a^	−28.90 ± 1.41 ^a^	−41.9	69.2%
		2%	4513 ± 617 ^a^	−24.28 ± 1.42 ^a^	−40.3	59.3%
		3%	4668 ± 455 ^a^	−24.28 ± 1.26 ^a^	−39.8	61.8%
	AL-Z	1%	9588 ± 2903 ^bcd^	−38.69 ± 4.43 ^bcd^	−48.4	71.1%
		2%	8537 ± 3620 ^cd^	−40.23 ± 2.83 ^cd^	−44.2	72.4%
		3%	6447 ± 451 ^d^	−41.36 ± 5.86 ^d^	−40.3	66.8%
	*p*-values					
	Starch		<0.001 *	<0.001 *	-	-
	Concentration		0.012 *	0.012 *	-	-
	Starch: Concentration		0.013 *	0.014 *	-	-
P3	AL-0%		4913 ± 328^a^	−35.16 ± 0.92^a^	−48.0	71.3%
	AL-N	1%	6791 ± 2084 ^ab^	−41.01 ± 1.61 ^ab^	−48.1	60.6%
		2%	5744 ± 40 ^ab^	−41.48 ± 1.65 ^ab^	−48.1	69.0%
		3%	6045 ± 1936 ^ab^	−38.00 ± 1.75 ^a^	−48.2	70.5%
	AL-Z	1%	7981 ± 2676 ^ab^	−40.66 ± 1.88 ^ab^	−47.8	69.4%
		2%	8401 ± 1882 ^ab^	−40.86 ± 5.03 ^ab^	−48.3	72.1%
		3%	11,694 ± 3883 ^b^	−45.36 ± 0.46 ^b^	−48.2	66.7%
	*p*-values					
	Starch		0.021 *	0.092	-	-
	Concentration		0.037 *	<0.001 *	-	-
	Starch: Concentration		0.215	0.043 *	-	-

** Samples with the same letter do not have a statistically significant difference. Samples with a different letter have significant differences. * Effects are statistically significant (*p*-value < 0.05).

## Data Availability

The supporting data can be found in Supplementary Materials, where spectrums taken in this study and figures about the materials synthesis are available.

## Supplementary Materials

The following supporting information can be downloaded at: https://www.mdpi.com/article/10.3390/polym14153184/s1, Table S1: Element weight composition of polyurethane composites from energy dispersive X-ray spectroscopy analysis. Figure S1: Synthesis of zwitterionic starch after (A) twelve hours of reaction, (B) neutralization, precipitation, and three wash with methanol, (C) smashing in methanol, and (D) filtration; Figure S2: 1H RMN spectrum of DCAPS; Figure S3: FTIR spectra of AL-N and AL-Z; Figure S4: AL-Z synthesis replicate (A) 1, (B) 2, (C) 3, and (D) 4; Figure S5: TGA of AL-N, AL-Z and DCPAS; Figure S6: FTIR spectra of (A) P1, (B) P2, and (C) P3 of polyurethane composites; Figure S7: TGA of polyurethane (A) without fillers, (B) P1, (C) P2, and (D) P3 composites; Figure S8: Loss factor of polyurethane composites P1 (A-B), P2 (C-D), and P3 (E-F); Figure S9: DSC of polyurethane (A) P1, (B) P2, and (C) P3 composites; Figure S10: XRD spectra of polyurethane composite P1 (A-B), P2 (C-D), and P3 (E-F).
