# Supplementary material for: Influence of Starch on the Structure–Properties Relationship in Polyethylene Glycol/Polycaprolactone Diol Polyurethanes
Source: Polymers (Basel). 2022 Aug 4;14(15):3184. doi: 10.3390/polym14153184 (PMC9371100; doi:10.3390/polym14153184)
Supplement: Supplementary file 1 [file polymers-14-03184-s001.zip › polymers-1847142-supplementary.pdf]

Supplementary material

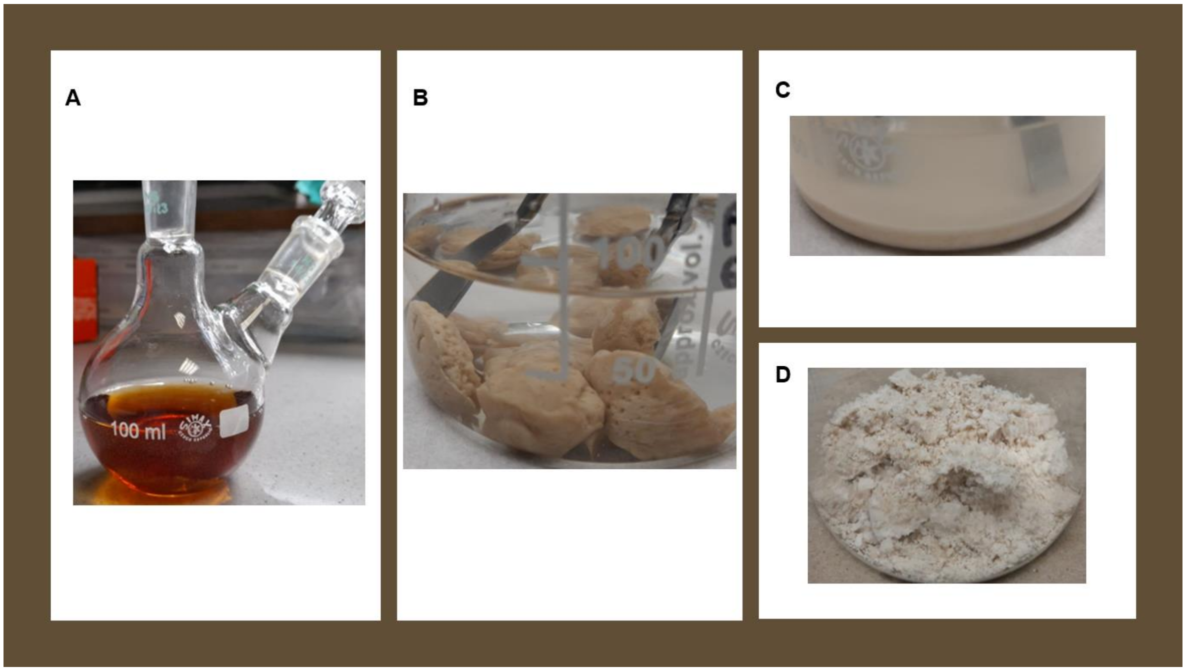

Figure S1. Synthesis of zwitterionic starch after (A) twelve-hour of reaction, (B) neutralization, precipitation, and three wash with methanol, (C) smash in methanol, and (D) filtration.

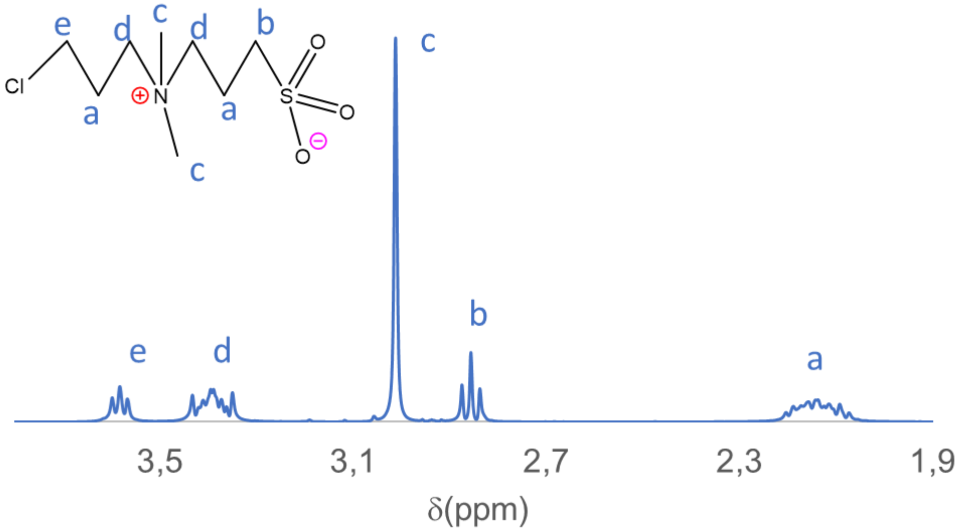

Figure S2. ^{1}H RMN spectrum of DCAPS

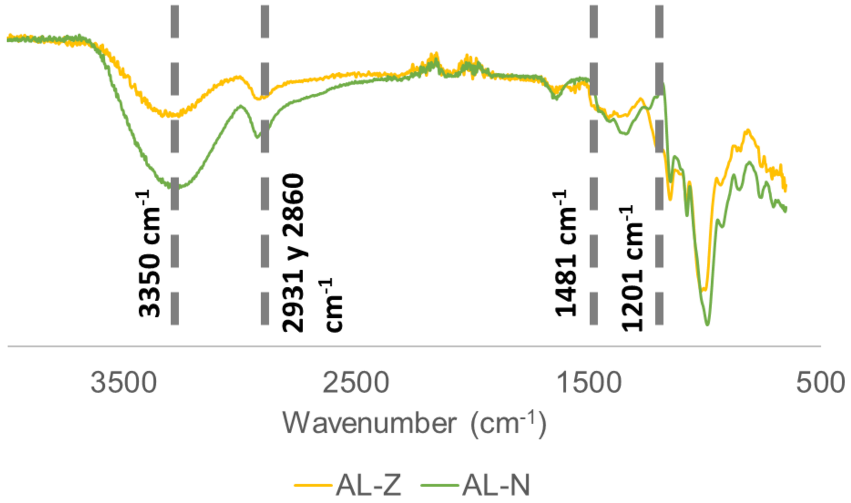

Figure S3. FTIR spectra of AL-N and AL-Z

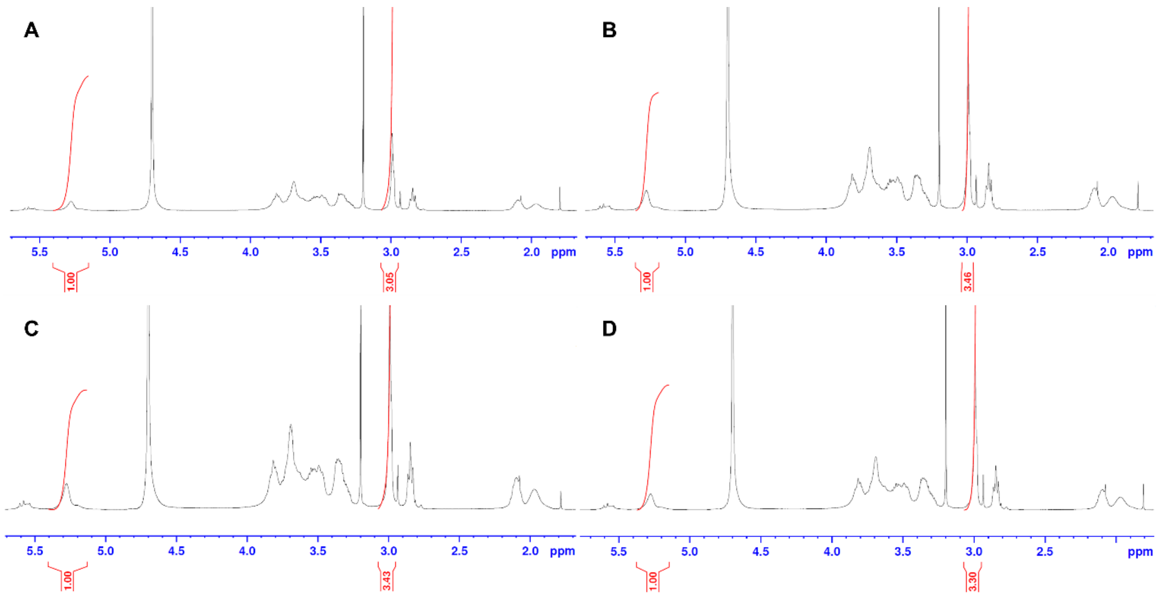

Figure S4. AL-Z synthesis replicate (A) 1, (B) 2, (C) 3, and (D) 4

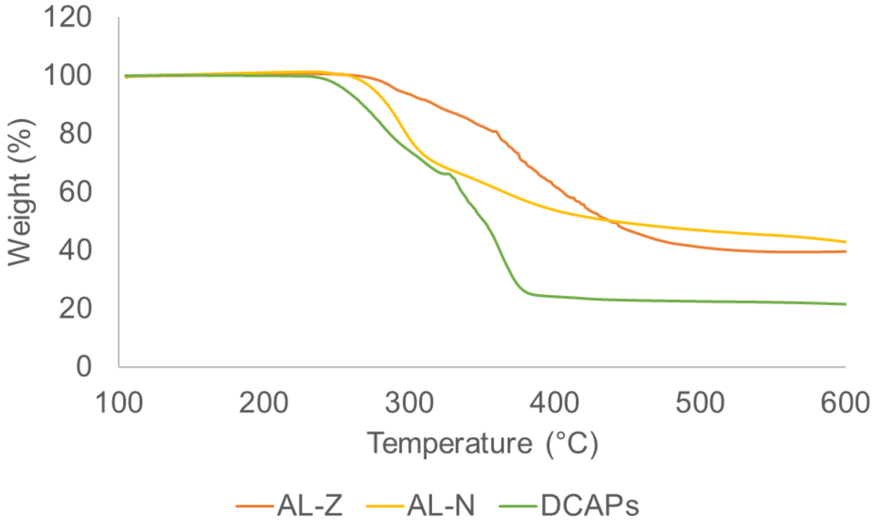

Figure S5. TGA of AL-N, AL-Z and DCPAS

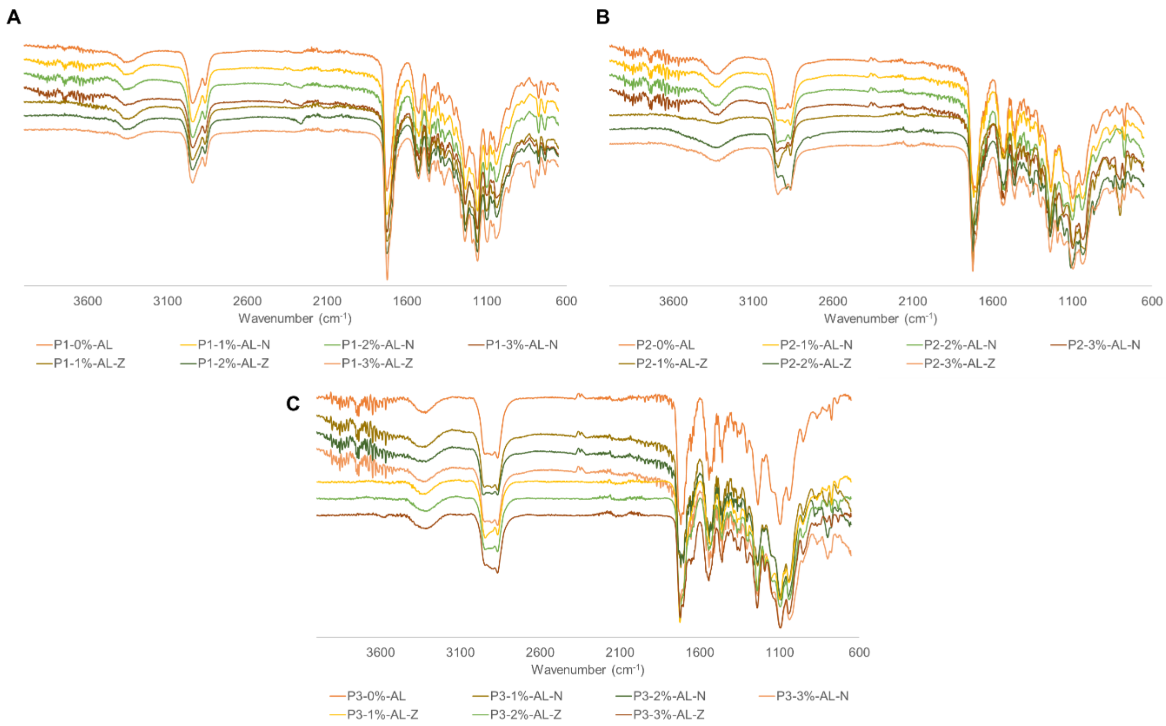

Figure S6. FTIR spectra of (A) P1, (B) P2, and (C) P3 of polyurethane composites

Table S 1. Element weight composition of polyurethane composites from energy dispersive X-ray spectroscopy analysis.

PU	Starch	Concentration	Zone	Element Wt%		
				C	O	S
		AL-N		71.05	28.95	0
		AL-Z		66.01	28.52	4.02
P1		AL-0%		87.95	12.05	0
	AL-N	3%	Matrix	85.61	14.39	0
			Filler	80.37	19.63	0
	AL-Z	3%	Matrix	85.73	12.56	0
			Filler	79.11	19.22	1.20

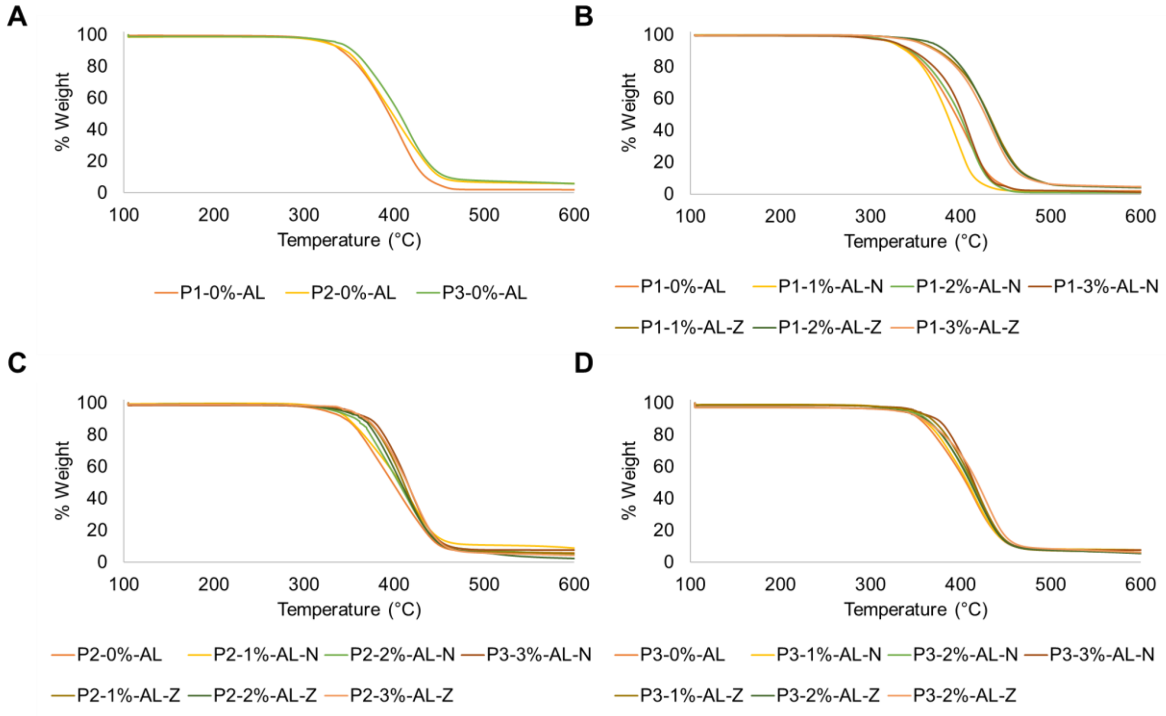

Figure S7. TGA of polyurethane (A) without fillers, (B) P1, (C) P2, and (D) P3 composites

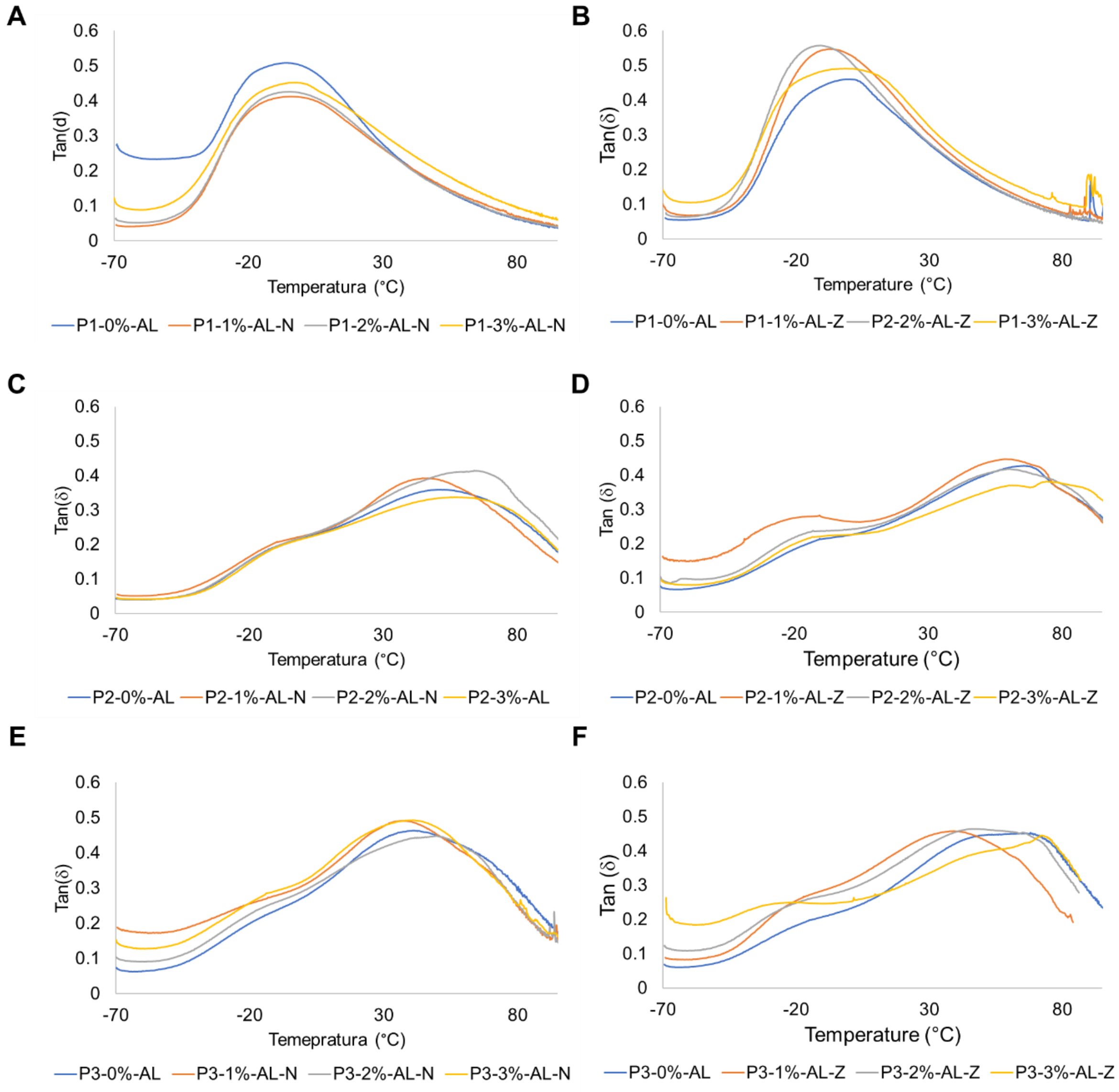

Figure S8. Loss factor of polyurethane composites P1 (A-B), P2 (C-D), and P3 (E-F)

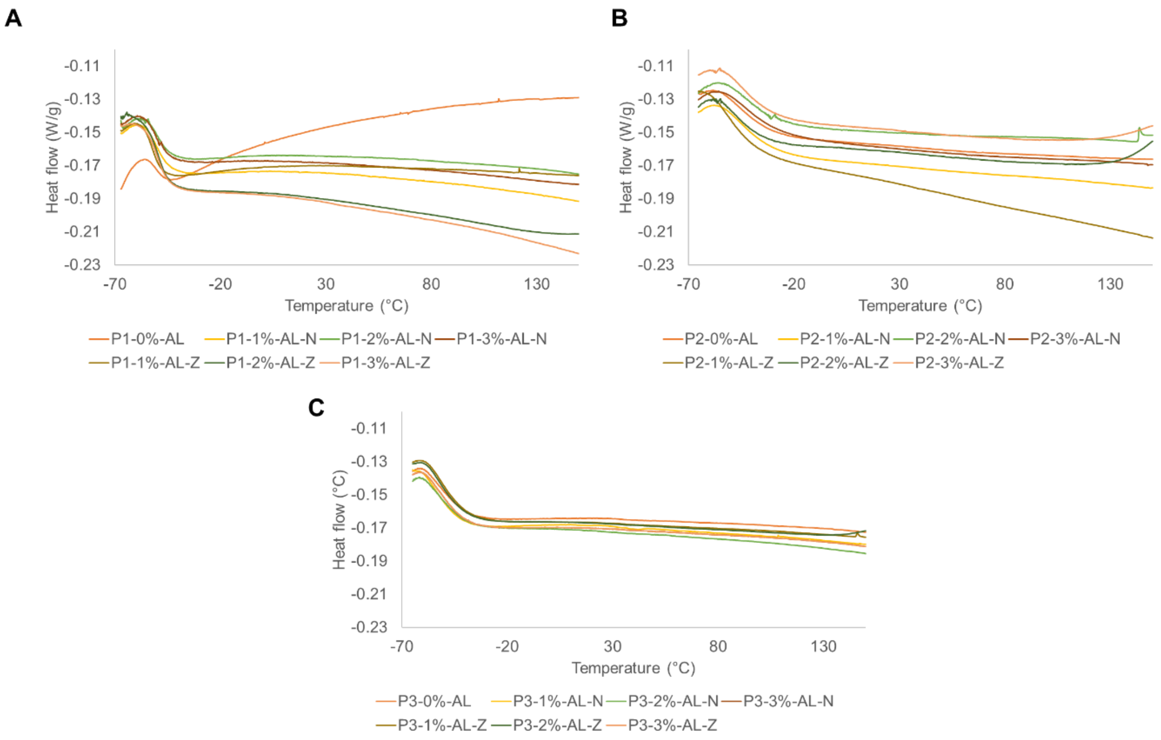

Figure S9. DSC of polyurethane (A) P1, (B) P2, and (C) P3 composites

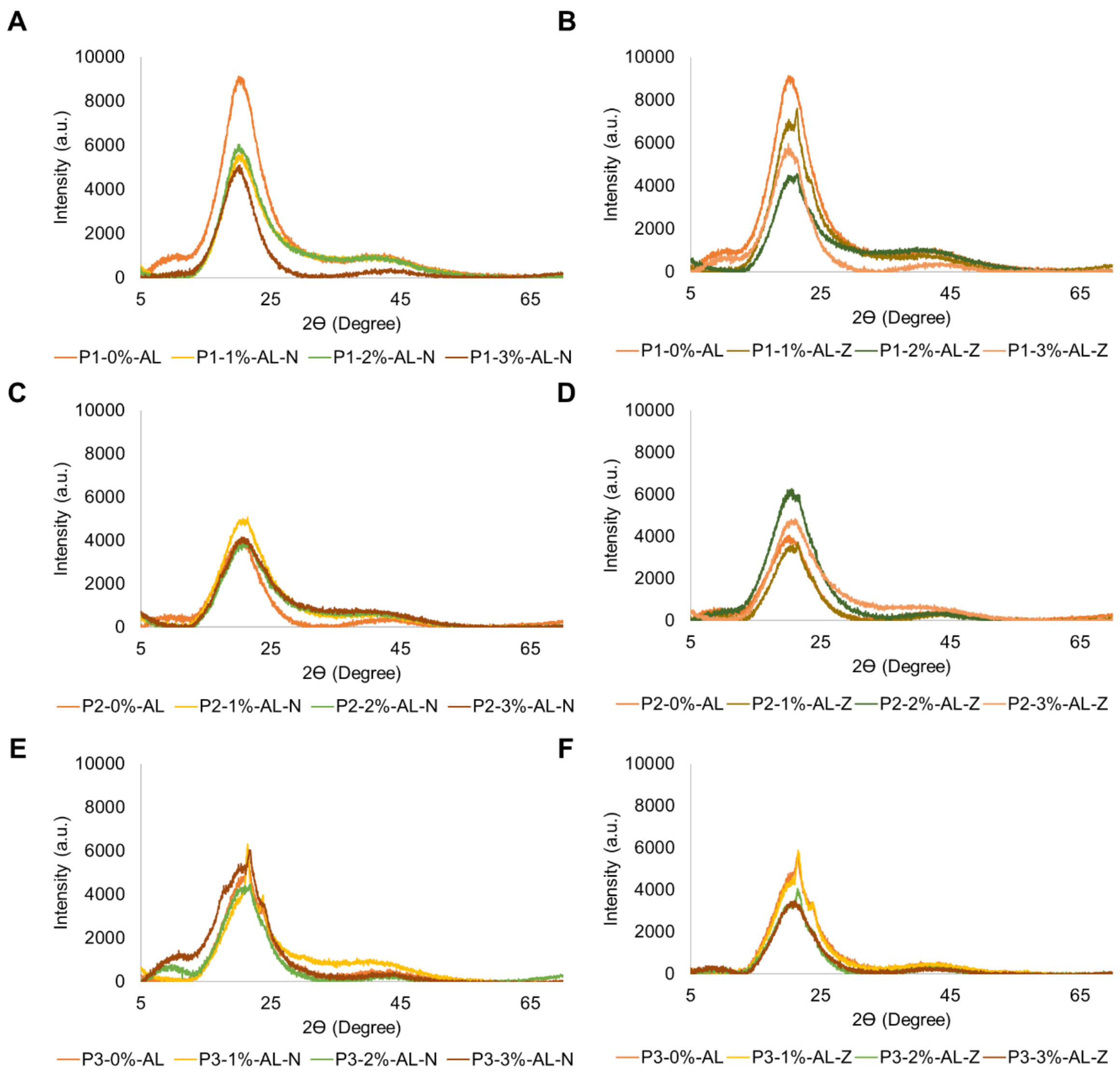

Figure S10. XRD spectra of composite polyurethane P1 (A-B), P2 (C-D), and P3 (E-F)
